# Establishment of Mouse Embryonic Stem Cell-Derived Erythroid Progenitor Cell Lines Able to Produce Functional Red Blood Cells

**DOI:** 10.1371/journal.pone.0001544

**Published:** 2008-02-06

**Authors:** Takashi Hiroyama, Kenichi Miharada, Kazuhiro Sudo, Inaho Danjo, Naoko Aoki, Yukio Nakamura

**Affiliations:** Cell Engineering Division, RIKEN BioResource Center, Tsukuba, Ibaraki, Japan; Texas Tech University Health Sciences Center, United States of America

## Abstract

**Background:**

The supply of transfusable red blood cells (RBCs) is not sufficient in many countries. If erythroid cell lines able to produce transfusable RBCs in vitro were established, they would be valuable resources. However, such cell lines have not been established. To evaluate the feasibility of establishing useful erythroid cell lines, we attempted to establish such cell lines from mouse embryonic stem (ES) cells.

**Methodology/Principal Findings:**

We developed a robust method to obtain differentiated cell lines following the induction of hematopoietic differentiation of mouse ES cells and established five independent hematopoietic cell lines using the method. Three of these lines exhibited characteristics of erythroid cells. Although their precise characteristics varied, each of these lines could differentiate in vitro into more mature erythroid cells, including enucleated RBCs. Following transplantation of these erythroid cells into mice suffering from acute anemia, the cells proliferated transiently, subsequently differentiated into functional RBCs, and significantly ameliorated the acute anemia. In addition, we did not observe formation of any tumors following transplantation of these cells.

**Conclusion/Significance:**

To the best of our knowledge, this is the first report to show the feasibility of establishing erythroid cell lines able to produce mature RBCs. Considering the number of human ES cell lines that have been established so far, the intensive testing of a number of these lines for erythroid potential may allow the establishment of human erythroid cell lines similar to the mouse erythroid cell lines described here. In addition, our results strongly suggest the possibility of establishing useful cell lines committed to specific lineages other than hematopoietic progenitors from human ES cells.

## Introduction

RBC transfusion was the first established transplantation procedure in clinical history, and is a common and indispensable clinical procedure. However, the supply of transfusable RBCs is insufficient in many countries. Thus, there is interest in the development of in vitro procedures for the generation of functional RBCs from hematopoietic stem and/or progenitor cells present in bone marrow or umbilical cord blood [1]–[3]. Human ES cells possess the potential to produce various differentiated cells able to function in vivo and thus represent another promising resource to produce functional RBCs.

Hematopoietic cells including cells of the erythroid lineage have been generated from mouse [4]–[7], non-human primate [8]–[10], and human ES cells [11]–[16]. We have recently established a method to culture hematopoietic cells derived from non-human primate ES cells long term in vitro [17]. The efficiency of generation of erythroid progenitors and/or RBCs varies based on the methods and ES cell lines used. Even with optimal experimental procedures and the most appropriate ES cell line, however, the generation of abundant RBCs directly from primate ES cells is a time-consuming process [17]. If human erythroid progenitor cell lines were established that could produce transfusable and functional RBCs efficiently, they would represent a much more useful resource to produce RBCs than ES cell lines.

Several mouse and human erythroid cell lines have been established. However, to the best of our knowledge, there is no cell line that can efficiently differentiate into enucleated RBCs. It is generally difficult to establish hematopoietic cell lines from adult hematopoietic stem or progenitor cells, since these somatic cells are quite sensitive to DNA damage and are unable to maintain the length of telomere repeats on serial passage [18]. By contrast, ES cells are quite resistant to DNA damage and maintain telomere length on serial passage [18]. Therefore, we speculated that these characteristics of ES cells may be advantageous for the establishment of cell lines, since differentiated cells derived from ES cells may retain such characteristics. In addition, mouse cells tend to immortalize more readily than human cells, as has been shown to be the case following the induction of pluripotent stem cell lines from somatic cells [19]–[22]. Hence, we attempted to evaluate the feasibility of establishing hematopoietic cell lines, erythroid cell lines in particular, from mouse ES cells.

## Results and Discussion

### Establishment of erythroid progenitor cell lines from mouse ES cells

To induce differentiation of hematopoietic cells from mouse ES cells, we cultured the latter cells using OP9 cells as feeder cells [5], [6], [23] in the presence of specific factors (Table 1). OP9 cells were used not only for induction of hematopoietic differentiation but also for establishment of cell lines in the early phase of long term culture of the induced hematopoietic cells (Table 1). In most cases, the induced cells failed to proliferate within two months of the initial induction of differentiation from ES cells (Table 2). Induced cells that could proliferate continuously for approximately two months (60 days) were subsequently cultured in the absence of OP9 cells and in the presence of hematopoietic humoral factors (Table 1). Cells that could proliferate in the absence of OP9 cells were cultured further. All established cell lines (Table 2) acquired independency from OP9 cells within three months of the initial induction of differentiation from ES cells. Approximately four months after the initial induction of differentiation of the cells, we evaluated the factors that were essential for the proliferation of each cell line (Table 1). After this evaluation, each cell line was cultured in the presence of these essential factors with medium changes every two or three days.

**Table 1:** Culture protocol.

Phase	Culture period	Feeder cells	Attached cells	Detached cells	Specific factors used
I	Day 0	OP9 cells	(Start)	(Start)	VEGF, IGF-II
II	Day 4	Change to new cells	Discarded^ a^	Re-cultured^ b^	SCF, EPO, IL-3, Dex
II	Day 7	No change	Remained^ c^	Re-cultured^ d^	SCF, EPO, IL-3, Dex
II	Day 10∼^e^	Change or No change^ f^	Remained or Discarded^ g^	Re-cultured^ h^	SCF, EPO, IL-3, Dex
III	Day 60∼^i^	(-) j	(-) k	Re-cultured^ h^	SCF, EPO, IL-3, Dex
IV	Day 120∼^l^	(-) j	(-) k	Re-cultured^ h^	Essential factors^ l^

To induce hematopoiesis, 5×10^5^ ES cells were cultured on feeder cells with cytokines in two 100 mm-dishes, with 2.5×10^5^ ES cells per dish. Phase I∼IV, four different phases of culture. Attached cells and Detached cells, the cells derived from ES cells and attached to feeder cells or detached from feeder cells. VEGF, vascular endothelial growth factor. IGF-II, insulin-like growth factor-II. SCF, stem cell factor. EPO, erythropoietin. IL-3, interleukin-3. Dex, dexamethasone.

a, the attached cells were discarded together with the used feeder cells. b, the detached cells collected from two dishes were cultured again on new OP9 cells in a 100 mm-dish. c, the attached cells were cultured further without any treatment. d, all detached cells collected from a dish were cultured again. e, medium changes were performed twice a week. f, when the attached cells reached approximately 80% confluence, feeder cells were changed to new OP9 cells. g, when the feeder cells were changed to new cells, the attached cells were discarded together with the used feeder cells. h, all detached cells collected from a dish were cultured again, or a portion of detached cells were cultured again and other detached cells were subjected to analyses or discarded. i, approximately as of Day 60 we started to try the culture in the absence of feeder cells using a portion of the detached cells, simultaneously continuing the culture in the presence of feeder cells as the Phase II culture. j, no feeder cells were used in the Phase III and IV culture. k, the cells attached to the dish were barely detected. l, approximately as of Day 120 the essential factor(s) for proliferation was evaluated, and then each cell line was cultured in the presence of the essential factor(s) alone.

**Table 2:** The number of trials to establish cell lines and the number of established cell lines.

Method A			
Name of ES cell line	Number of trial	Number of established cell line	Designation
E14TG2a	10	1	MEDEP-E14
D3	3	0	
TT2	3	0	
BRC4	7	0	
BRC5	10	1	MEDEP-BRC5
BRC6	6	1	MEDMC-BRC6
BRC7	6	0	
NTES2	6	1	MEDMC-NT2
Method B			
Name of ES cell line	Number of trial	Number of established cell line	Designation
BRC4	4	1	MEDEP-BRC4
BRC5	2	0	
BRC6	4	0	
BRC7	2	0	

Method A, the method described in Table 1. Method B, the use of IL-3 was excluded from Method A through all procedures.

In addition to the method described in Table 1 (Method A), we developed Method B in which the use of IL-3 was excluded from Method A through all procedures (Table 2). We attempted long term culture of 63 lines, 51 lines with Method A and 12 lines with Method B, and succeeded in establishing five independent immortalized cell lines, 4 lines with Method A and 1 line with Method B (Table 2). These five cell lines could proliferate continuously for more than one year. Morphological and flow cytometric analyses suggested that three of these lines were erythroid in nature, as shown below, and that the other two lines were mast cell-like (Figures S1 and S2). We designated the erythroid cell lines MEDEP (mouse ES cell-derived erythroid progenitor line) and the mast cell-like cell lines MEDMC (mouse ES cell-derived mast cell line). MEDEP-E14, MEDEP-BRC4, and MEDEP-BRC5 were derived from E14TG2a, BRC4, and BRC5 mouse ES cell lines, respectively. The presence of IL-3 in the culture medium (Method A) may not be necessary for the establishment of erythroid cell lines, as we were able to establish one erythroid line, MEDEP-BRC4, following culture of the cells in the absence of IL-3 (Method B) (Table 2). MEDEP cells could proliferate from single cells following sorting by flow cytometry (data not shown).

**Figure 1:**
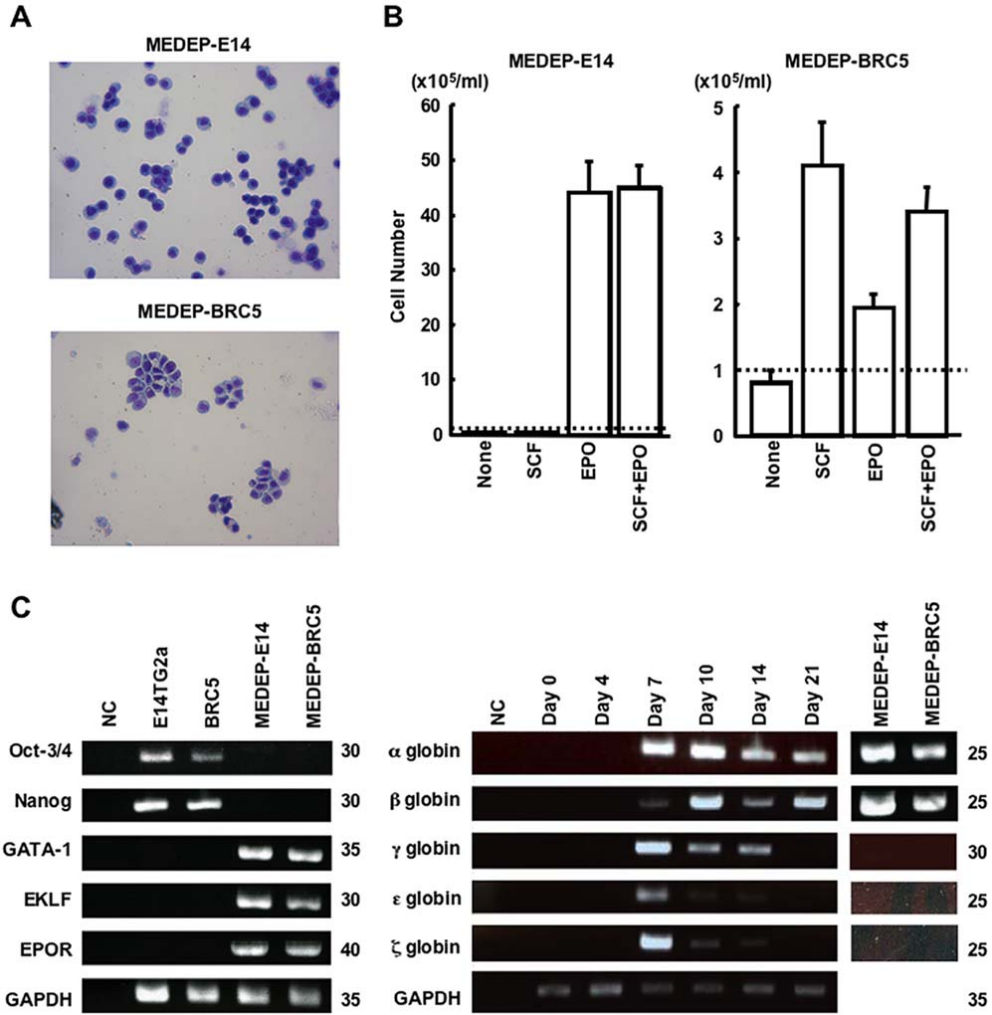
Characteristics of erythroid cell lines derived from mouse ES cells, MEDEP. (A) Morphology of two erythroid cell lines, MEDEP-E14 and MEDEP-BRC5. Wright-Giemsa staining. (B) Cytokine dependent proliferation. Cells (1×10^5^ cells/ml) were cultured in various conditions for three days. The added factor(s) is shown at the bottom. None, no specific factor. SCF, stem cell factor. EPO, erythropoietin. Broken line, the number of cells at the start of culture. Values are mean±S.D. Results shown are representative of several independent experiments performed at different time points after establishment of the cell lines. (C) RT-PCR analyses. Oct-3/4 and Nanog, transcription factors specific for ES cells. GATA-1 and EKLF (Erythroid Krüppel-like factor), transcription factors specific for erythroid cells. EPOR, erythropoietin receptor. GAPDH, glyceraldehyde-3-phosphate dehydrogenase. NC, negative control without cDNA. Day 0, E14TG2a cells before differentiation. Day 4, 7, 10, 14 and 21, the cells following induction of differentiation into hematopoietic cells from E14TG2a by the method described in Table 1 (Method A). The cycle numbers performed in each PCR are shown at the right. Results shown are representative of two independent experiments.

MEDEP-E14 and MEDEP-BRC5 cells retained morphological characteristics of erythroid cells (Figure 1A) and cytokine dependency (Figure 1B) after cloning. MEDEP-E14 and MEDEP-BRC5 were dependent on erythropoietin (EPO) and stem cell factor (SCF), respectively (Figure 1B). Although MEDEP-BRC5 appeared to respond to EPO (Figure 1B), it could not proliferate long term in the presence of EPO alone (data not shown). MEDEP-BRC4 also showed morphological characteristics of erythroid cells (Figure S3) and could proliferate most efficiently in the presence of SCF, EPO, and dexamethasone (Figure S3). The cytokine dependency of these cell lines has not changed for more than one year after the induction of their differentiation from ES cells.

RT-PCR analyses demonstrated that all MEDEP lines expressed genes specific for erythroid cells: GATA-1, EKLF (Erythroid Krüppel-like factor) and EPOR (erythropoietin receptor) (Figure 1C and Figure S4). In addition, all MEDEP lines expressed α- and β-globin, but not γ-, ε-, or ζ-globin (Figure 1C and Figure S4), indicating that they were adult and not primitive erythroid progenitor cells. Since the induction of definitive erythropoiesis, i.e., the induction of adult type erythroid cells, from mouse ES cells has previously been reported [6], all MEDEP lines appeared to be derived from adult type erythroid progenitor cells.

### In vitro differentiation of MEDEP

**Figure 2:**
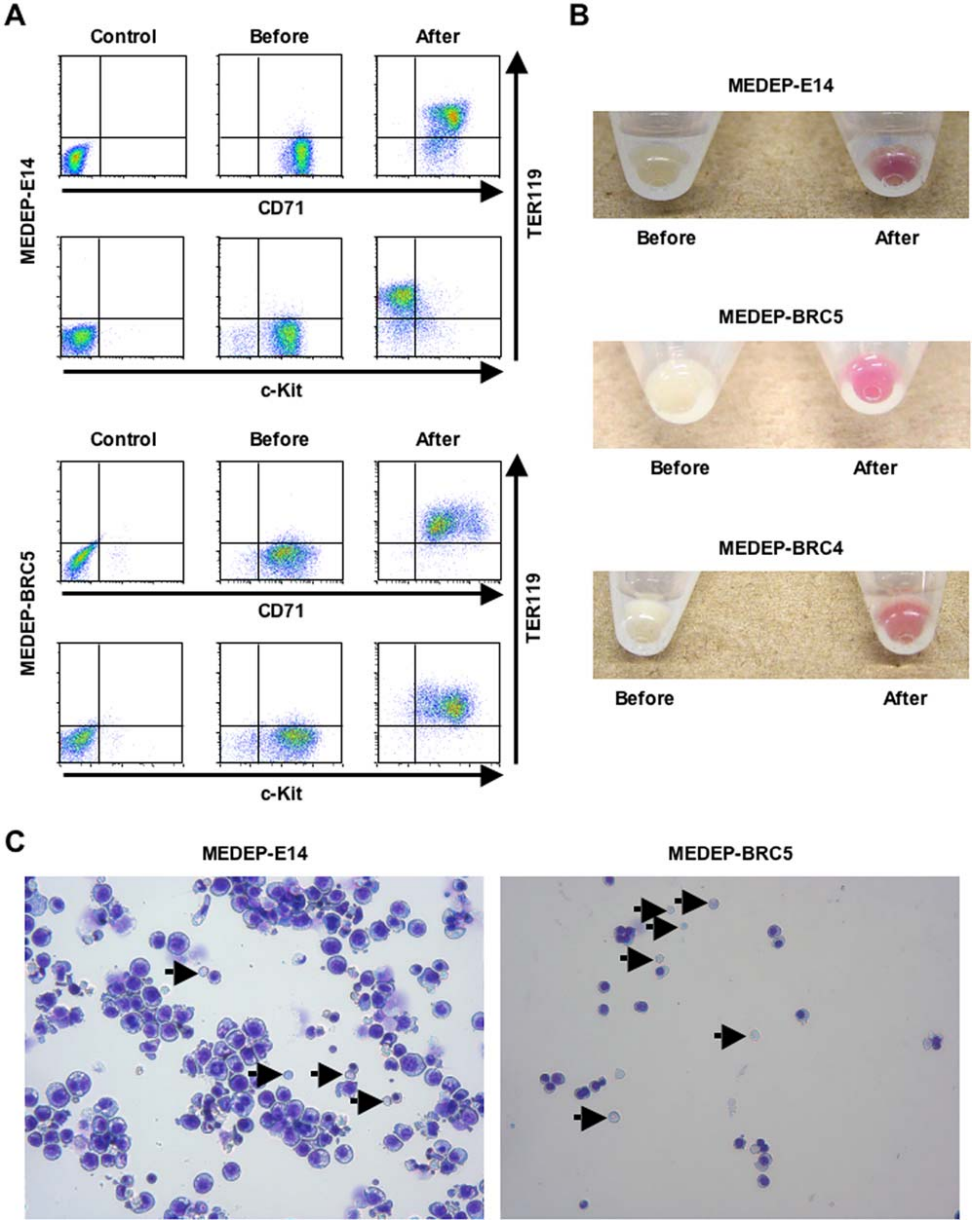
In vitro differentiation of MEDEP. The in vitro differentiation of MEDEP-E14 was performed by culture for two days after deprivation of erythropoietin (EPO). The in vitro differentiation of MEDEP-BRC5 was performed by culture for three days after deprivation of stem cell factor (SCF) and addition of EPO. (A) Flow cytometric analyses. Control, results with isotype controls. Before and After, the cells before and after in vitro differentiation. CD71, transferrin receptor. c-Kit, receptor for SCF. TER119, a cell surface antigen specific for mature erythroid cells. (B) Cell pellets before and after in vitro differentiation. The method for in vitro differentiation of MEDEP-BRC4 is described in Figure S4. (C) Morphology of the cells after in vitro differentiation. Arrows indicate enucleated red blood cells. (A–C) Results shown are representative of three independent experiments.

Next, we evaluated the potential of MEDEP cells to differentiate into more mature erythroid cells. We found that all MEDEP lines could differentiate into more mature erythroid cells by the following treatments: deprivation of EPO for MEDEP-E14 (Figure 2A); deprivation of SCF and addition of EPO for MEDEP-BRC5 (Figure 2A); and deprivation of SCF and dexamethasone and addition of EPO for MEDEP-BRC4 (Figure S4). EPO appeared to be necessary for MEDEP-BRC5 and MEDEP-BRC4 cells to maintain cell viability during the differentiation process (data not shown).

The three MEDEP lines exhibited differential expression of TER119 (a cell surface antigen specific for mature erythroid cells) and CD71 (transferrin receptor) (Figure 2A and Figure S4). For example, the expression of CD71 was slightly higher in MEDEP-E14 cells than in MEDEP-BRC5 cells (Figure 2A). TER119^−^CD71^−^ cells differentiate first to TER119^−^CD71^+^ cells, subsequently to TER119^++^CD71^+^ cells, and then finally to TER119^+^CD71^−^ cells [25]. Consistent with the differences in their cytokine dependency (Figure 1B), the MEDEPs appeared to represent different stages of erythroid differentiation. Despite these differences, following induction of differentiation in vitro by the methods described above, the expression of TER119 and CD71 in each of the MEDEP lines exhibited a pattern consistent with a more mature lineage (Figure 2A and Figure S4), indicating that each of the three lines was able to differentiate into a more mature lineage. At present, the cause of the variability between MEDEPs remains uncertain. However, these results clearly demonstrated that erythroid progenitor cells could be immortalized at different stages of their differentiation.

Notably, the vast majority of cells in each MEDEP line could differentiate into more mature cells, although each MEDEP line included cells possessing abnormal karyotypes (Figure S5). This result strongly suggested that the cells possessing abnormal karyotypes still retained the potential to differentiate into more mature erythroid cells. In general, most immortalized cell lines are not necessarily homogenous in karyotype and/or characteristic, even after cloning. The emergence of cells possessing different karyotypes and/or different characteristics is often observed following long term utilization of immortalized cell lines. Hence, periodical recloning and selection of cell lines is recommended to maintain their characteristics.

Following induction of differentiation in vitro, cell pellets appeared red while the cell pellets before differentiation appeared white (Figure 2B). In addition, the appearance of enucleated cells following differentiation was demonstrated by flow cytometric analysis using SYTO85 staining (Figure S6). Moreover, it was confirmed by morphological analysis that enucleated RBCs in addition to very mature erythroblasts were present following induction of differentiation (Figure 2C, arrows).

### In vivo proliferation and differentiation of MEDEP

**Figure 3:**
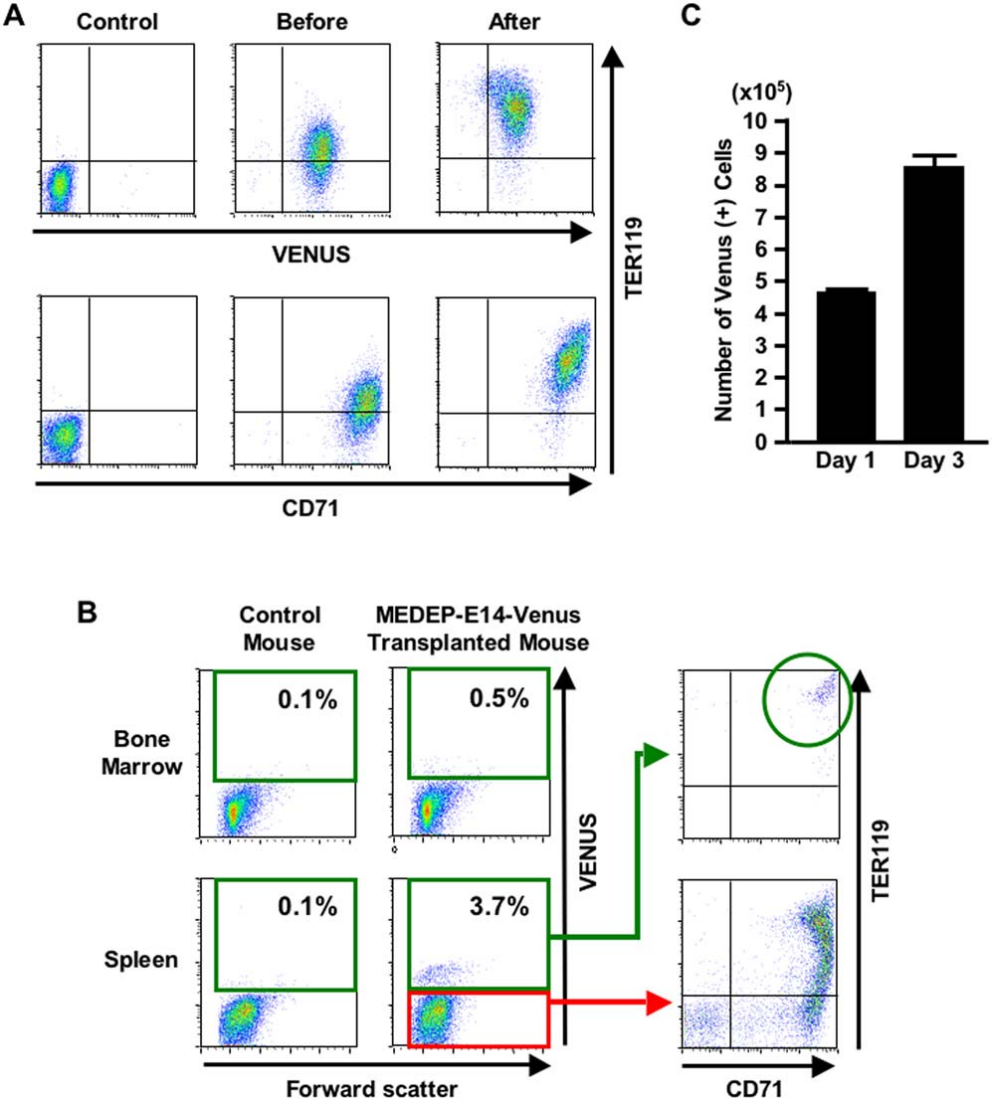
In vivo proliferation and differentiation of MEDEP. A transformant of MEDEP-E14 expressing Venus as a marker was established, MEDEP-E14-Venus. (A) The in vitro differentiation of MEDEP-E14-Venus was performed by culture for two days after deprivation of erythropoietin. Control, results with isotype controls. Before and After, the cells before and after in vitro differentiation. (B) In vivo differentiation of MEDEP-E14-Venus cells. Acute anemia was induced in an immuno-deficient mouse (NOD-SCID) and the next day MEDEP-E14-Venus cells (2×10^7^ cells/mouse) were transplanted into the anemic mouse. Three days after cell transplantation, bone marrow and spleen cells were subjected to flow cytometric analyses. Control mouse, NOD-SCID mouse without cell transplantation. The vast majority of Venus-positive cells in the spleen show differentiation into CD71^+^TER119^+^ mature erythroid cells. (A, B) CD71 and TER119, see legend of Figure 2A. Results shown are representative of three independent experiments. (C) In vivo proliferation of MEDEP-E14-Venus cells. Cell transplantation was performed as in (B). We determined the proportion (%) of Venus-positive cells and calculated the absolute number of Venus-positive cells in the spleen. Day 1 and Day 3, one day and three days following cell transplantation, respectively. Values are mean±S.D. (N = 3).

To evaluate the functional potential of MEDEP cells in vivo, we established a subline of MEDEP-E14 expressing Venus [26] as a marker, MEDEP-E14-Venus. Although the expression of TER119 was slightly higher in MEDEP-E14-Venus cells than in MEDEP-E14 cells (compare Figure 2A and Figure 3A), MEDEP-E14-Venus cells retained the ability to proliferate (data not shown) and differentiate into more mature erythroid cells in vitro (Figure 3A).

In general, the ablation of endogenous hematopoietic cells in mice is required to detect transplanted hematopoietic cells efficiently. Acute anemia induced by phlebotomy or hemolysis is commonly applied in the study of urgent erythropoiesis [25], [27]. Hence, we induced acute anemia in mice by intraperitoneal injection of phenylhydrazine, an inducer of hemolysis, and transplanted MEDEP-E14-Venus cells (2×10^7^ cells/mouse) 24 hours later. Three days after cell transplantation, Venus-positive cells were present in the bone marrow and spleen (Figure 3B). Since the spleen is the major organ supporting urgent erythropoiesis [25], the transplanted cells were observed more abundantly in the spleen than in the bone marrow (Figure 3B). Venus positive cells (the transplanted cells) demonstrated a phenotype consistent with differentiation into more mature erythroid cells (Figure 3B) compared to the phenotype of the cells just before transplantation (Figure 3A, before differentiation). Of note, MEDEP-E14-Venus cells differentiated into much more mature lineages in vivo than they did in vitro (compare Figure 3A and Figure 3B).

To evaluate whether transplanted cells can proliferate in vivo, we determined the proportion (%) of Venus-positive cells and calculated the absolute number of Venus-positive cells in the spleen in a cell transplantation experiment performed similarly to that shown in Figure 3B. The absolute number of Venus-positive cells was elevated approximately two fold at three days after cell transplantation compared to that at one day (Figure 3C). This result indicates that transplanted cells can proliferate in vivo. However, this proliferation was transient and the transplanted cells did not form a tumor in vivo, as shown below.

The expression of Venus in the transplanted cells decreased following the differentiation of them, i.e., the expression of Venus was lower in TER119^++^ cells than in TER119^+^ cells (Figure 3A). Thus, although we could not detect Venus-positive cells in peripheral blood (data not shown), it was highly likely due to the disappearance of Venus following the terminal differentiation. To confirm that MEDEP could differentiate into terminally-differentiated RBCs in vivo, we performed the following experiments.

### Increase of RBC number in mice suffering from acute anemia following transplantation of MEDEP

**Figure 4:**
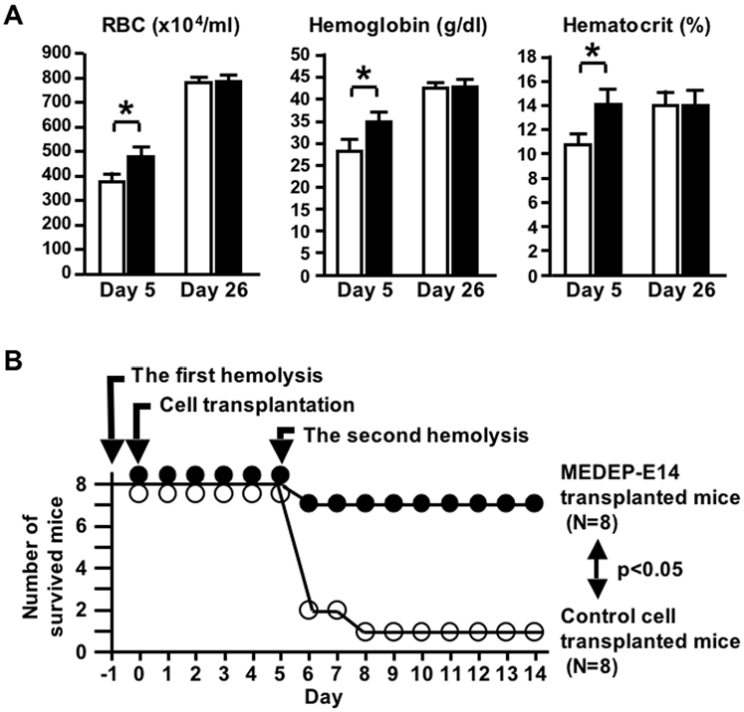
Amelioration of anemia by transplantation of MEDEP. (A) MEDEP-E14 cells (2×10^7^ cells/mouse) were transplanted into an immuno-deficient mouse (NOD-SCID) 24 hours after the induction of hemolysis by phenylhydrazine (60 mg/kg body weight) injection. Day 5 and Day 26, five and twenty-six days after cell transplantation. RBC, red blood cell. White bars (n = 10) and black bars (n = 14), the data obtained from the mice transplanted with control cells and MEDEP-E14 cells, respectively. Values are mean±S.D. * p<0.01 (by the Student's *t*-test) (B) Increased survival of mice transplanted with MEDEP cells following induction of severe acute anemia. MEDEP-E14 cells (2×10^7^ cells/mouse) were transplanted into an NOD-SCID mouse 24 hours following the first induction of hemolysis by phenylhydrazine (60 mg/kg body weight) injection. Five days following the cell transplantation, the second induction of hemolysis by phenylhydrazine (80 mg/kg body weight) injection was performed. Statistical analysis was performed using the chi-square test. (A, B) Control cell, mast cell line derived from mouse ES cells (MEDMC-NT2) (Figures S1 and S2). MEDMC-NT2 cells (2×10^7^ cells/mouse) were transplanted similarly as a control experiment.

MEDEP cells (2×10^7^ cells/mouse) were transplanted 24 hours after induction of acute anemia. As a control experiment, MEDMC cells (2×10^7^ cells/mouse) were transplanted into control mice. Since 2×10^7^ transplanted RBCs correspond to a mere 2 µl of transfused cells, the number of RBCs in transplanted mice will only increase if these transplanted MEDEP cells proliferate to some degree and differentiate into terminally-differentiated RBCs in vivo. Five days after the transplantation, the peripheral blood was subjected to a blood count. The transplantation of MEDEP-E14 significantly ameliorated anemia compared to the control (Figure 4A). The data obtained from the mice transplanted with control cells (Figure 4A) did not differ significantly from the data obtained from the anemic mice that were not transplanted with any cells (data not shown).

Since the RBC count in peripheral blood reflects the number of enucleated cells, whereas erythroblasts (nucleated cells) are counted as white blood cell (WBC), the increased number of RBC observed in mice transplanted with MEDEP cells (Figure 4A) indicated that the transplanted MEDEP cells could differentiate into enucleated cells very efficiently. Since the life span of RBCs is approximately 50 days in the mouse, it is highly likely that the RBCs produced from the transplanted MEDEP cells accumulated in the transplanted mouse.

Increases in mean corpuscular volume (MCV), mean corpuscular hemoglobin (MCH), and MCH concentration (MCHC) (Figure S7) are commonly observed in the recovery phase of acute anemia [25]. In addition, an increase in the number of WBC observed in the recovery phase of acute anemia (Figure S7) is due to the presence of erythroblasts in the peripheral blood, since erythroblasts are counted as WBC by the automatic counter [25]. Given that there was no difference in MCV, MCH and MCHC levels between the two groups in the recovery phase of acute anemia (Figure S7), RBCs derived from MEDEP cells in vivo appeared to possess characteristics quite similar to those derived from erythroid progenitor cells in the host mice. Twenty-six days after transplantation (27 days after the induction of acute anemia), all mice had recovered from the anemia and there were no differences in the blood counts of the two groups (Figure 4A and Figure S7).

The transplantation of MEDEP-E14-Venus and MEDEP-BRC5 cells also ameliorated anemia compared to the control (Table 3). However, the transplantation of MEDEP-BRC5 cells appeared to be less effective for amelioration of anemia than MEDEP-E14 cells (compare Figure 4A and Table 3). Given that the in vitro proliferation activity of MEDEP-BRC5 cells was lower than that of MEDEP-E14 cells (Figure 1B), the in vivo proliferation activity of MEDEP-BRC5 cells might have also been lower than that of MEDEP-E14 cells. In addition, hemoglobin synthesis in MEDEP-BRC5 might have been less efficient than that in MEDEP-E14 (Figure 2B and Table 3).

**Table 3:** Blood count after induction of acute anemia and cell transplantation.

	RBC	Hb	Ht	MCV	MCH	MCHC	WBC	Platelet
Experiment A								
Day 5								
MEDEP-E14-Venus (N = 3)	502.0±30.4*	14.1±0.7*	36.7±1.0*	73.3±3.9	28.1±0.6	38.4±1.3	680.3±86.1	97.1±14.6
MEDMC-NT2 (N = 3)	401.0±11.4	10.8±0.3	29.2±0.8	72.8±0.6	26.9±0.2	37.0±0.5	783.3±53.2	98.0±3.7
Day 26								
MEDEP-E14-Venus (N = 3)	761.3±31.0	15.0±0.3	41.7±1.2	54.7±0.7	19.7±0.3	36.0±0.3	17.0±1.7	118.7±11.6
MEDMC-NT2 (N = 3)	772.0±25.3	15.1±0.5	41.4±1.8	53.7±0.3	19.5±0.3	36.5±0.3	12.7±5.7	124.3±9.3
Experiment B								
Day 5								
MEDEP-BRC5 (N = 10)	562.7±22.4*	13.0±1.1	34.7±1.4*	61.6±1.2	23.0±1.1	37.4±1.7	150.7±36.1	75.5±8.0
MEDMC-BRC6 (N = 10)	506.9±31.7	12.1±0.7	32.3±1.5	63.8±2.5	23.8±1.2	37.4±2.5	216.4±100.5	86.1±19.2
Day 26								
MEDEP-BRC5 (N = 10)	933.5±34.4	14.9±1.2	49.0±2.3	52.5±1.5	15.9±1.0	30.4±1.3	72.5±17.3	95.1±6.9
MEDMC-BRC6 (N = 10)	922.7±26.1	14.8±1.0	48.7±1.2	52.8±1.2	16.0±1.0	30.3±1.4	78.3±17.7	105.0±10.3

Experiment A, MEDEP-E14-Venus and MEDMC-NT2 (2×10^7^ cells/mouse) were transplanted into immuno-deficient mice (NOD-SCID) 24 hours following the induction of hemolysis by phenylhydrazine (60 mg/kg body weight) injection. Experiment B, MEDEP-BRC5 and MEDMC-BRC6 (2×10^7^ cells/mouse) were transplanted into C57BL/6 mice 24 hours following the induction of hemolysis by phenylhydrazine (80 mg/kg body weight) injection. Day 5 and Day 26, five and twenty-six days after cell transplantation. RBC, red blood cell, ×10^4^/µl. Hb, hemoglobin, g/dl. Ht, hematocrit, %. MCV, mean corpuscular volume, fl. MCH, mean corpuscular hemoglobin, pg. MCHC, MCH concentration, g/dl. WBC, white blood cell, ×10^2^/µl. Platelet, ×10^4^/µl.

* p<0.01 (by the Student's *t*-test)

Immunogenicity of human ES cell derivatives is one of the potential obstacles to the clinical use of them [28], [29]. In fact, transplanted MEDEP cells could not ameliorate acute anemia in mouse strains other than those from which each individual line was derived or in immuno-deficient mice, suggesting immunological rejection by the heterologous strains. Hence, if human erythroid cell lines could be established, the clinical application of such cell lines may require many cell lines expressing the different major histo-compatibility (MHC) antigens.

### Lack of tumorigenicity of MEDEP

Approximately three months (82 days) after transplantation, Venus-positive cells were absent from the bone marrow and spleen of mice transplanted with MEDEP-E14-Venus cells (Figure S8). In addition, although we observed all other transplanted mice up to 6 months after transplantation, no tumor was observed in MEDEP-transplanted mice or MEDMC-transplanted control mice (data not shown). Furthermore, subcutaneous transplantation of MEDEP cells (2×10^7^ cells/injection site) did not give rise to any tumors, whereas subcutaneous transplantation of the same number of parent ES cells led to the formation of a teratoma (data not shown).

Nevertheless, if human erythroid cell lines were established, the tumorigenic potential of those cell lines should be thoroughly analyzed prior to use in the clinic [30], [31]. It may be advisable to engineer such cells so that they could be eliminated should a malignant phenotype arise for any reason [32].

### RBCs derived from MEDEP are functional in vivo

To confirm that the RBCs derived from the transplanted MEDEP cells are functional in vivo, we monitored the response of transplanted mice to a second induction of hemolysis. Similar to the experiments shown in Figure 4A and Table 3, a prior induction of hemolysis and subsequent cell transplantation were performed. A second induction of hemolysis was performed five days after the cell transplantation (Figure 4B). Analysis of blood count was not performed at any time point in this experiment, because collection of peripheral blood would affect the results. We observed that one out of eight mice in the group transplanted with MEDEP-E14 cells died while seven out of eight mice in the group transplanted with control cells (MEDMC-NT2) died (Figure 4B). The mice that did not receive any transplanted cells demonstrated a mortality similar to that of mice transplanted with control cells (data not shown). This result was consistent with the increased RBC number five days after cell transplantation (Figure 4A). In other words, this result indicated that RBCs derived from MEDEP cells were functional in vivo and that mice transplanted with MEDEP cells could survive the induction of severe acute anemia following a second induction of hemolysis.

### Concluding Remarks

At present, we cannot precisely describe the exact mechanism underlying the establishment of differentiated cell lines from ES cells. Nevertheless, our results clearly indicate that we can reproducibly obtain useful erythroid cell lines from mouse ES cells. Given that differentiation strategies developed for mouse ES cells can differ from methods applied to human ES cells in many cases [33], the method we developed here may not be applied to human ES cells directly and some modifications may be necessary. However, considering the number of human ES cell lines that have been established so far, we believe that these human ES cell lines, at least in part, should exhibit the potential to produce erythroid cell lines. In addition, our results strongly suggest the possibility of establishing useful cell lines committed to specific lineages other than hematopoietic progenitors from human ES cells.

The induction of terminally differentiated cells that no longer proliferate should enable clinical applications of ES cell derivatives without an associated risk of tumorigenicity. For example, RBCs lack nuclei following terminal differentiation, and thus are highly unlikely to exhibit tumorigenicity in vivo. As such, even if the original ES cells and/or their derivatives possessed abnormal karyotypes and/or genetic mutations, they may nonetheless be useful for clinical applications, provided that they can produce enucleated RBCs. In fact, although our MEDEP lines included many cells possessing abnormal karyotypes (Figure S5), the vast majority of cells in each cell line could nevertheless differentiate into mature erythroid cells (Figure 2A), including enucleated cells (Figure 2C).

We showed a model of transplantation therapy using MEDEPs in this study as an application of ES cell-derivatives. On the other hand, methods to produce enucleated RBCs abundantly from human hematopoietic stem cells in vitro have recently been reported [2], [3]. Therefore, once appropriate erythroid cell lines have been established, it would be possible to use such methods to produce enucleated RBCs from such cell lines in vitro. The establishment of a human erythroid cell line lacking the genes to produce the A, B, and RhD antigens would be a universal resource for clinical application, since the cell line produces O/RhD(-) RBCs which are transfusable into all individuals in theory.

## Materials and Methods

### Mouse ES cell lines

Mouse ES cell lines, E14TG2a and D3, were obtained from the American Type Culture Collection (ATCC; Monassas, VA, USA). E14TG2a and D3 were derived from 129/Ola and 129/Sv+c/+p mice, respectively. Other mouse ES cell lines were obtained from the Cell Engineering Division of RIKEN BioResource Center (Tsukuba, Ibaraki, Japan). TT2 was derived from F1 C57BL/6 and CBA mice. BRC4, BRC5, BRC6, and BRC7 were derived from C57BL/6 mice. NTES-2 was established following transfer of nuclei of cells derived from 129 mice into oocytes.

ES cells were maintained in an undifferentiated state on mouse embryonic fibroblasts (MEFs) in a 1∶1 mixture of Dulbecco's modified Eagle's medium (DMEM; SIGMA, St Louis, MO, USA) and Ham's nutrient mixture F-12 (SIGMA) supplemented with 1,000 U/ml leukemia inhibitory factor (LIF; CHEMICON International Inc., Temecula, CA, USA), 0.1 mM non-essential amino acids, 2 mM L-glutamine, 0.1 mM 2-mercaptoethanol, and 20% KnockOut™ Serum Replacement (KSR; Invitrogen, Carlsbad, CA, USA). Before MEFs were used as feeder cells, they were irradiated with γ-rays (50 Gy).

### Establishment of hematopoietic cell lines from mouse ES cell lines

The feeder cell line, OP9 [23], used to induce hematopoietic differentiation of ES cells [5], [6] was obtained from the Cell Engineering Division of RIKEN BioResource Center and was cultured in Minimum Essential Medium-α (MEM-α; SIGMA) containing 20% fetal bovine serum (FBS; SIGMA). Before OP9 cells were used as feeder cells, they were irradiated with γ-rays (50 Gy).

The basal medium used throughout long term culture was Iscove's modified Dulbecco's medium (IMDM; Invitrogen) containing the following materials: 15% FBS (SIGMA); 10 µg/ml bovine insulin, 5.5 µg/ml human transferrin, and 5 ng/ml sodium selenite (ITS liquid MEDIA supplement; SIGMA); 50 µg/ml ascorbic acid (SIGMA); 0.45 mM α-monothioglycerol (SIGMA); 100 unit/ml penicillin, 100 µg/ml streptomycin, and 2 mM L-glutamine (PSQ; Invitrogen).

Specific factors used were as follows: mouse vascular endothelial growth factor (VEGF, 20 ng/ml; R&D systems, Minneapolis, MN, USA), mouse insulin-like growth factor-II (IGF-II, 200 ng/ml; R&D systems), mouse stem cell factor (SCF, 50 ng/ml; R&D systems), human erythropoietin (EPO, 5 unit/ml; KIRIN Brewery Company, Tokyo, Japan), mouse interleukin-3 (IL-3, 10 ng/ml; R&D systems), and Dexamethasone (Dex, 10^−6^ M; SIGMA).

We developed two different methods to establish hematopoietic cell lines from mouse ES cells, Method A and Method B. Method A is described in Table 1. In Method B, the use of IL-3 was excluded from Method A through all procedures.

Viable cell number was assessed using an automated cell counter and an assay based on the trypan blue dye exclusion method, ViCell™ (BECKMAN COULTER, Fullerton, CA, USA). Morphology of the established cell lines was analyzed after Wright-Giemsa (Sysmex International, Kobe, Japan) staining.

### Reverse transcription-polymerase chain reaction (RT-PCR)

A semi-quantitative RT-PCR was performed as described previously [24]. PCR was carried out with recombinant Taq polymerase (TaKaRa Bio Inc., Otsu, Shiga, Japan). Cycling parameters were as follows: denaturation at 94°C for 30 sec, annealing at 55°C for 30 sec, and extension at 72°C for 30 sec. PCR products were separated on 1.5% agarose gels and visualized by ethidium bromide staining. Amplification of the gene encoding glyceraldehyde-3-phosphate dehydrogenase (GAPDH) was used as an internal control in the PCR.

The sequences of the PCR primers were as follows: for mouse Oct-3/4, the sense primer was 5′-ACC CAG GCC GAC GTG GGG CT-3′ and the antisense primer was 5′-TTC TGG CGC CGG TTA CAG AAC CA-3′ (365-bp PCR product); for mouse Nanog, the sense primer was 5′-TAC CTC AGC CTC CAG CAG AT-3′ and the antisense primer was 5′-CCT CCA AAT CAC TGG CAG-3′ (460-bp PCR product); for mouse GATA-1, the sense primer was 5′-ACA GGT CAC TAC CTG TGC AAT GCC-3′ and the antisense primer was 5′-CCT GAC AGT ACC ACA GGT CCT AG-3′ (463-bp PCR product); for mouse EKLF (Erythroid Krüppel-like factor), the sense primer was 5′-TAT GGG CTG CTG TCG GGA TAC CC-3′ and the antisense primer was 5′-TCA GAG GTG ACG CTT CAT GTG CAG -3′ (507-bp PCR product); for mouse EPOR (erythropoietin receptor), the sense primer was 5′-ATC CAT ATC AAT GAA GTA GTG CTC-3′ and the antisense primer was 5′-CCA CAG CTG GAA GTT ACC CTT GTG-3′ (513-bp PCR product); for mouse α-globin, the sense primer was 5′-CTC TCT GGG GAA GAC AAA AGC-3′ and the antisense primer was 5′-GGT GGC TAG CCA AGG TCA CCA-3′ (334-bp PCR product); for mouse β (β-major)-globin, the sense primer was 5′-GAT GCT GAG AAG TCT GCT GTC-3′ and the antisense primer was 5′-CTG GAA GGC AGC CTG TGC AGC-3′ (381-bp PCR product); for mouse γ (β-H1)-globin, the sense primer was 5′-CTC AAG GAG ACC TTT GCT CA-3′ and the antisense primer was 5′-AGT CCC CAT GGA CTC AAA GA-3′ (265-bp PCR product); for mouse ε-globin, the sense primer was 5′-GGA GAG TCC ATT AAG AAT CTA-3′ and the antisense primer was 5′-CTG TGA ATT CAT TGC CGA AGT-3′ (157-bp PCR product); for mouse ζ-globin, the sense primer was 5′-GCT CAG GCC GAG CCC ATT GG-3′ and the antisense primer was 5′-TAG CGG TAC TTC TCA GTC AG-3′ (371-bp PCR product); and for mouse GAPDH (glyceraldehyde-3-phosphate dehydrogenase), the sense primer was 5′-GTC TTC ACC ACC ATG GAG AAG-3′ and the antisense primer was 5′-GCC ATC CAC AGT CTT CTG GGT-3′ (270-bp PCR product).

### Flow cytometry

Cells were stained with monoclonal antibodies (MoAbs) and analyzed by FACS Calibur (BD Biosciences, San Jose, CA, USA) or sorted by FACSVantage SE (BD Biosciences). The following MoAbs were purchased from BD Biosciences: a fluorescein isothiocyanate (FITC)-conjugated MoAb against mouse CD71 (transferrin receptor), a phycoerythrin (PE)-conjugated MoAb against TER119 (a cell surface antigen specific for mature erythroid cells), an allophycocyanin (APC)-conjugated MoAb against mouse c-Kit (receptor for stem cell factor), a PE-conjugated MoAb against mouse CD71, and a biotin-conjugated MoAb against TER119. APC-conjugated streptavidin (BD Biosciences) was used to detect biotin-conjugated MoAb. To distinguish nucleated and enucleated cells, we used the SYTO85 nuclear stain (Invitrogen). Cell viability was monitored by propidium iodide (SIGMA) staining. Flow cytometry data were analyzed using FlowJo (Tree Star Inc., Ashland, OR, USA) analysis software. Morphology of the sorted cells was analyzed following Wright-Giemsa (Sysmex International) staining.

### Mice

Eight-week-old female NOD/shi-scid Jic and C57BL/6NCrj mice were purchased from CLEA Japan (Tokyo, Japan) and Charles River Laboratories Japan (Yokohama, Kanagawa, Japan), respectively. Mice were used within a week of delivery in all experiments. All experimental manipulations of mice were approved by the Institutional Animal Care and Use Committee of the RIKEN Tsukuba Institute.

### Induction of acute anemia in mice

Acute anemia was induced by hemolysis following intraperitoneal injection of phenylhydrazine (Wako, Osaka, Japan), a chemical inducer of hemolysis, at doses of 60 mg/kg body weight for NOD/shi-scid Jic mice and 80 mg/kg body weight for C57BL/6NCrj mice. The second induction of hemolysis shown in Figure 4B was performed at a dose of 80 mg/kg body weight in NOD/shi-scid Jic mice.

### Transplantation of cells

Cells (2×10^7^ cells/mouse) were injected into the tail vein of an 8-week-old female mouse.

### Blood count

Peripheral blood samples were obtained from the retro-orbital venous plexus. We estimated the number of white blood cells, red blood cells, and platelets, in addition to the hemoglobin concentration and hematocrit, using an automated Celltac α MEK-6358 counter (NIHON-KODEN, Tokyo, Japan).

### Statistical analysis

All statistical analyses were performed using Statcel (OMS company, Saitama, Japan) analysis software. As for the two-sample *t*-test, the data were analyzed by the F test for variance followed by the Student's *t*-test.

## Supporting Information

Figure S1 (10.19 MB TIF)

Figure S2 (10.19 MB TIF)

Figure S3 (10.19 MB TIF)

Figure S4 (10.19 MB TIF)

Figure S5 (10.20 MB TIF)

Figure S6 (10.19 MB TIF)

Figure S7 (10.19 MB TIF)

Figure S8 (10.19 MB TIF)
