# Supplementary figures and images for: Establishment of Mouse Embryonic Stem Cell-Derived Erythroid Progenitor Cell Lines Able to Produce Functional Red Blood Cells
Source: PLoS One. 2008 Feb 6;3(2):e1544. doi: 10.1371/journal.pone.0001544 (PMC2212133; doi:10.1371/journal.pone.0001544)

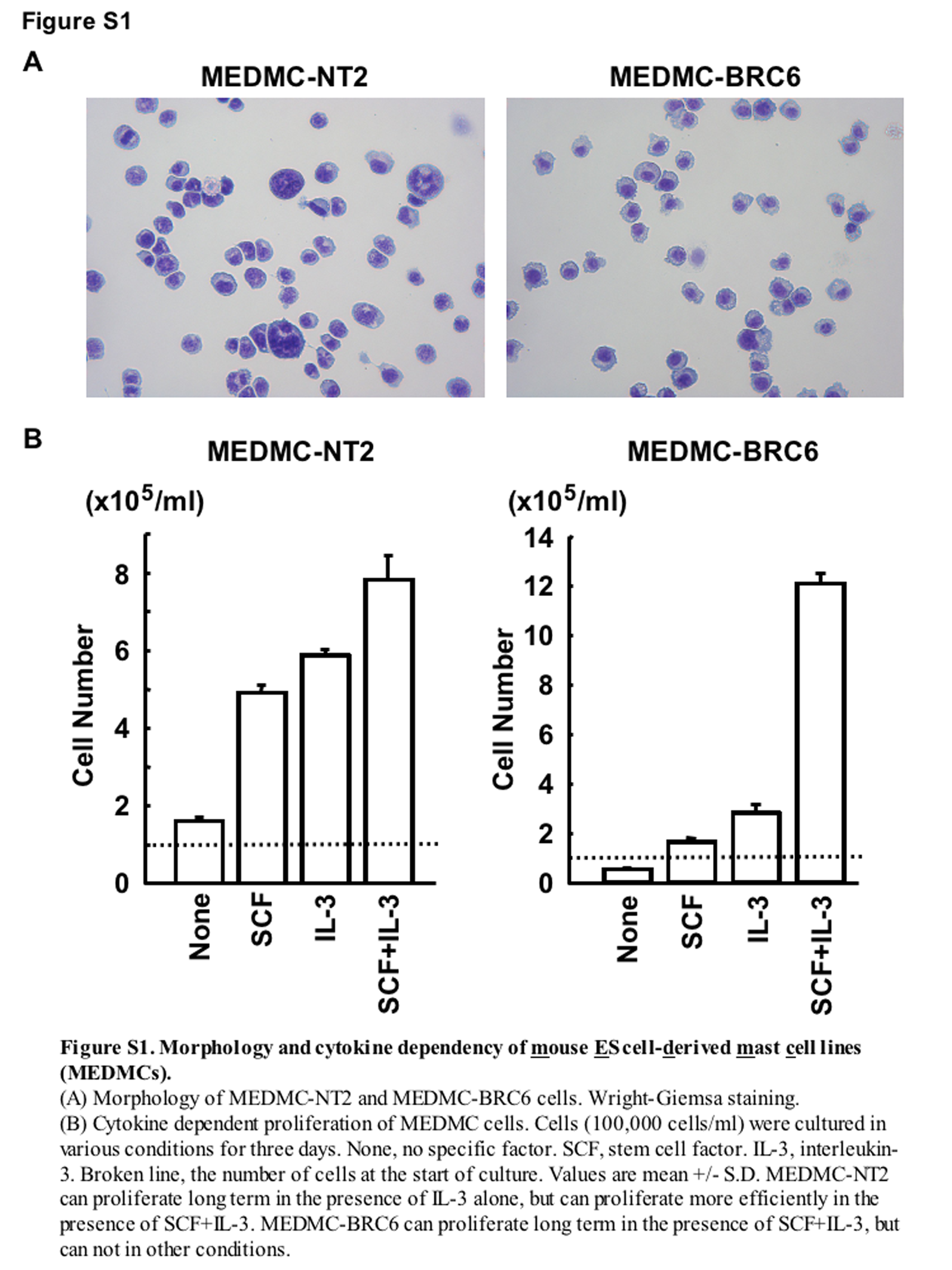

Supplement: Figure S1 — (10.19 MB TIF) [file pone.0001544.s001.tif]

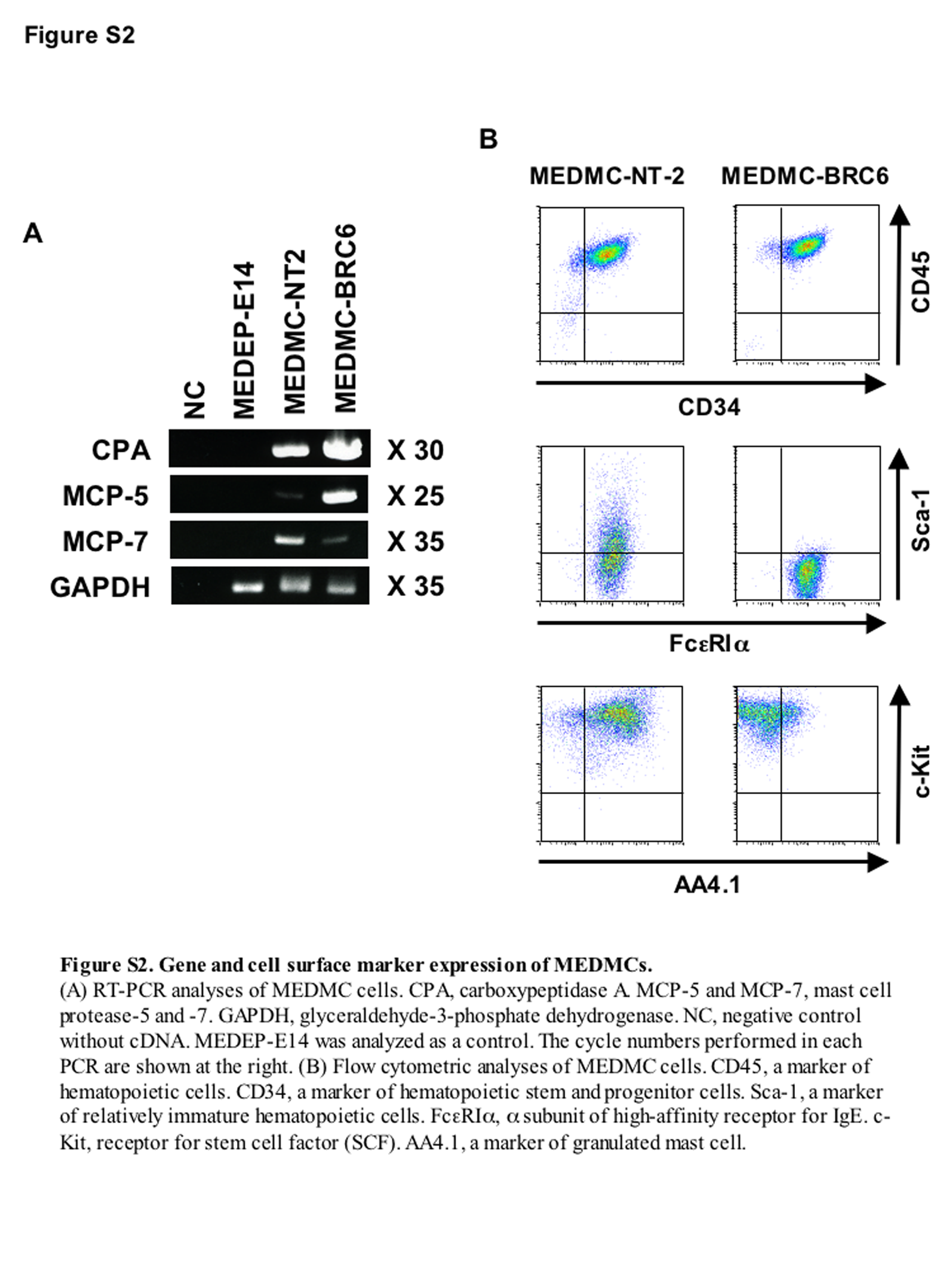

Supplement: Figure S2 — (10.19 MB TIF) [file pone.0001544.s002.tif]

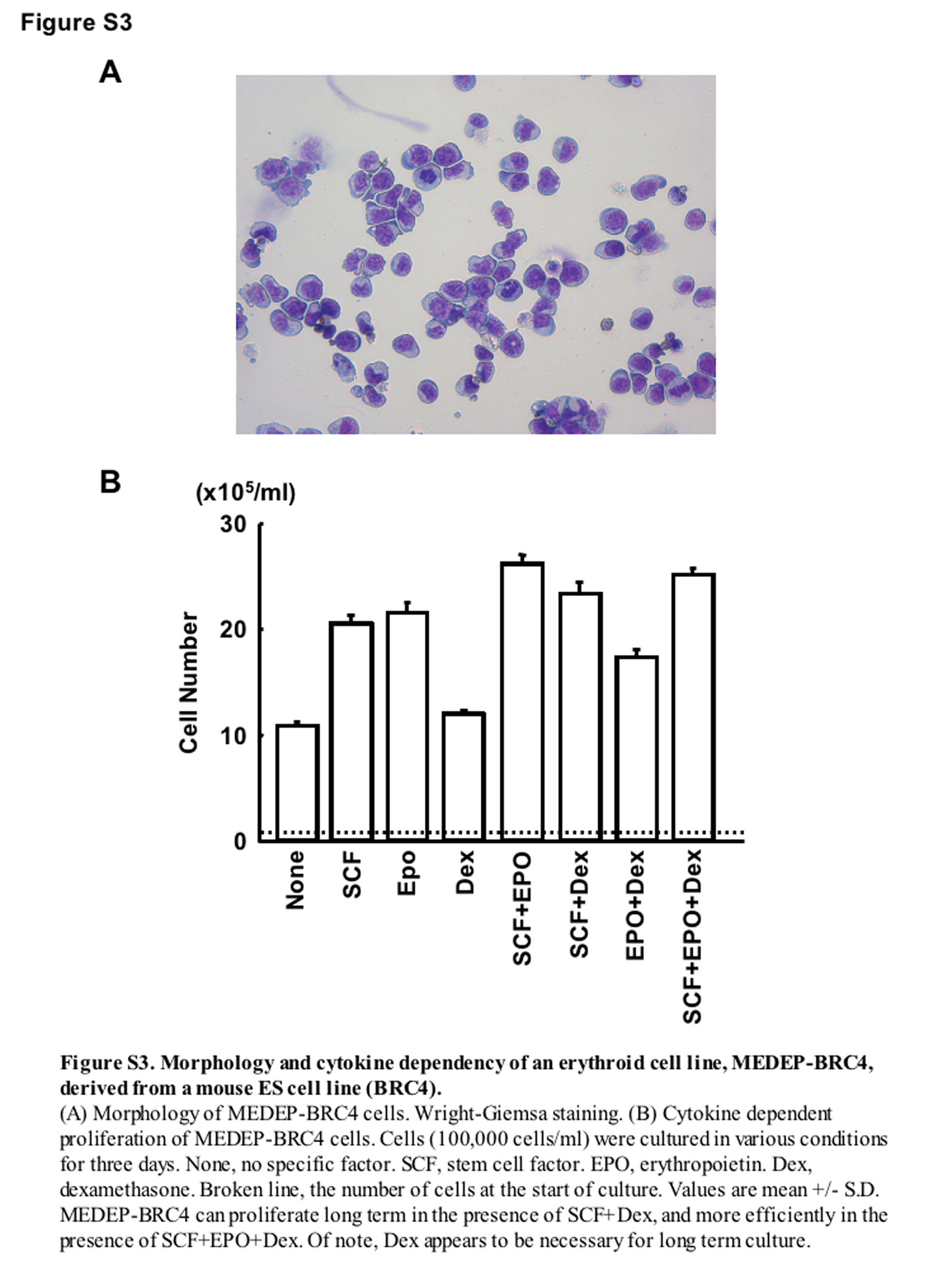

Supplement: Figure S3 — (10.19 MB TIF) [file pone.0001544.s003.tif]

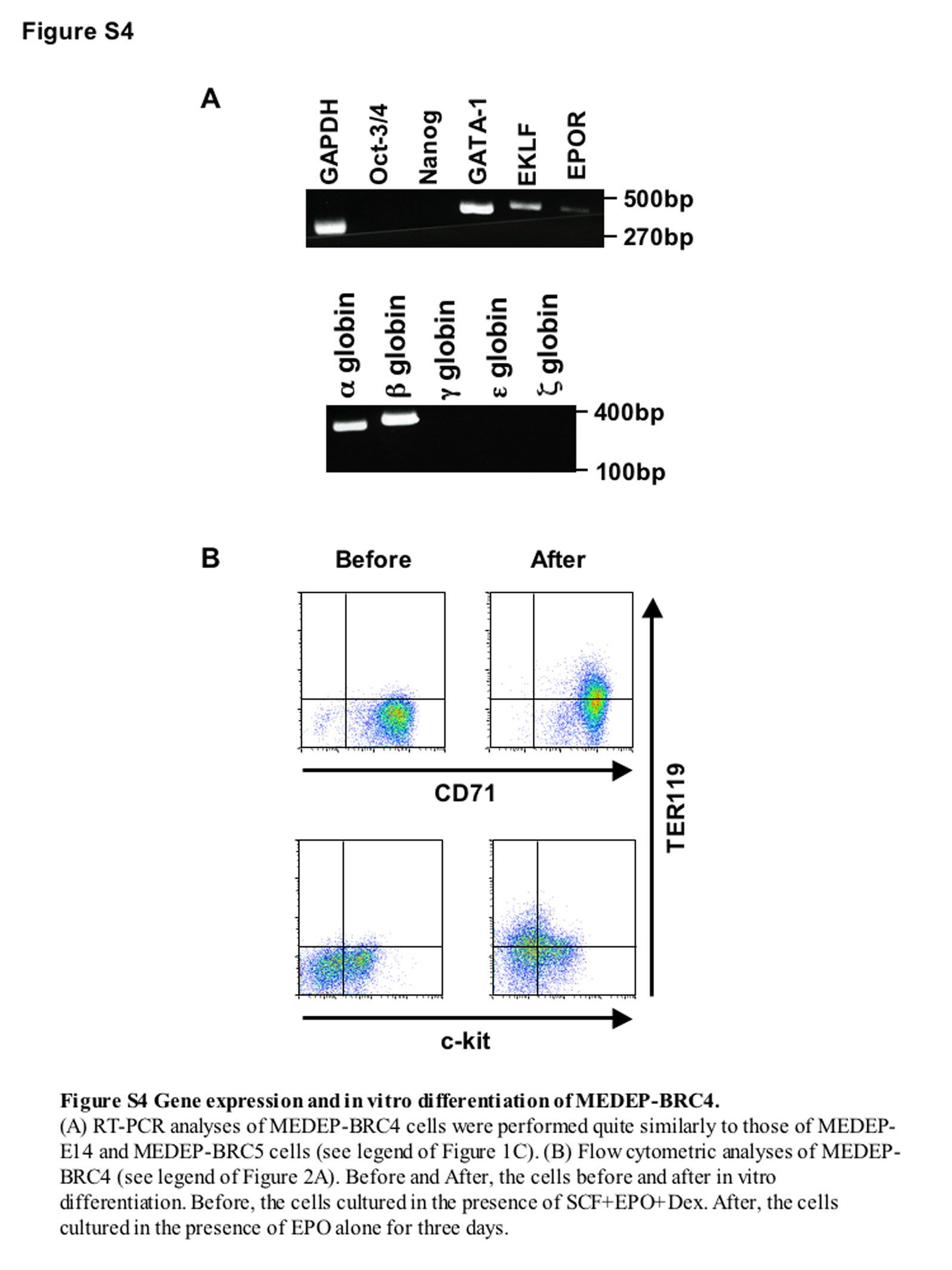

Supplement: Figure S4 — (10.19 MB TIF) [file pone.0001544.s004.tif]

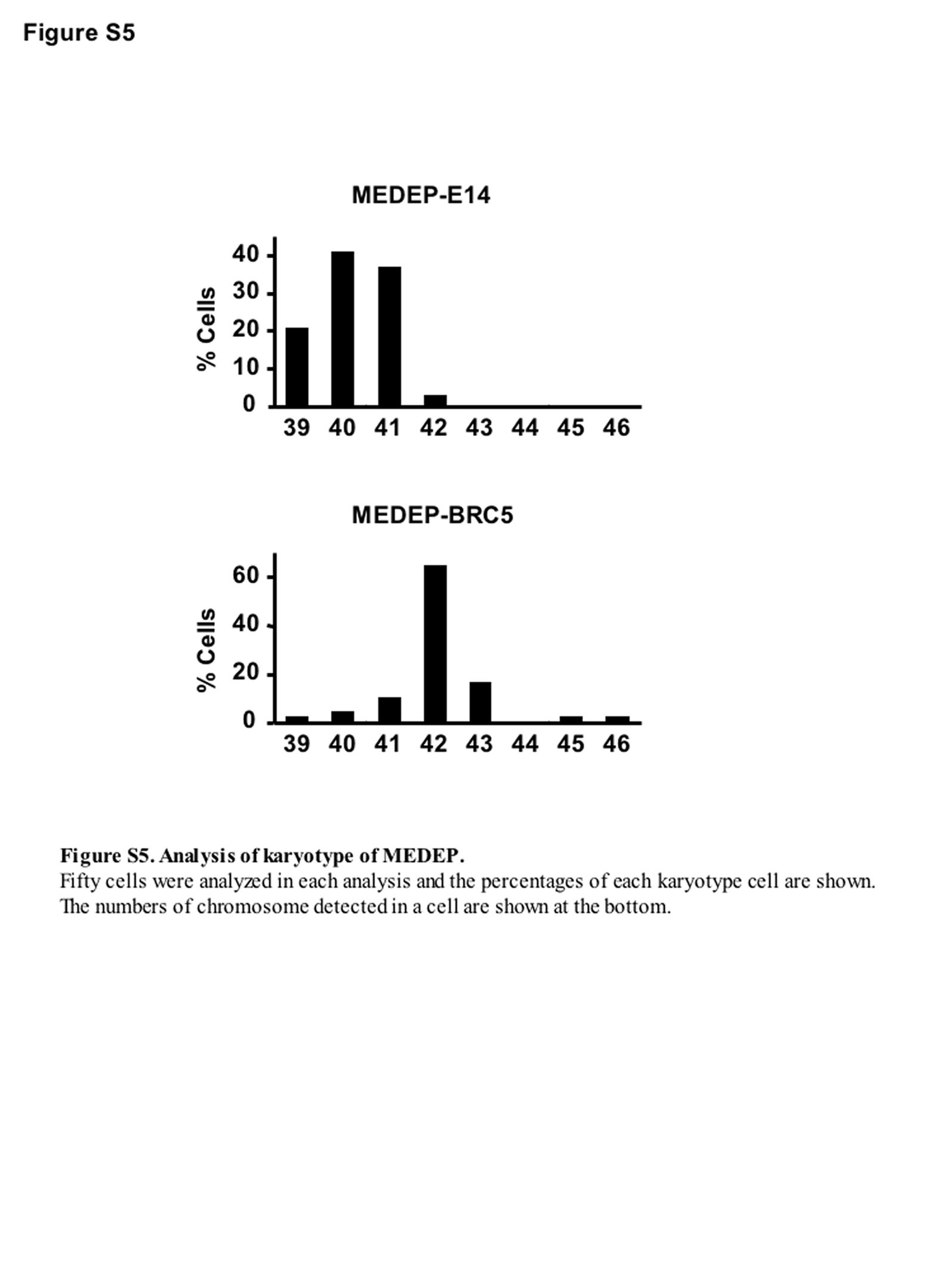

Supplement: Figure S5 — (10.20 MB TIF) [file pone.0001544.s005.tif]

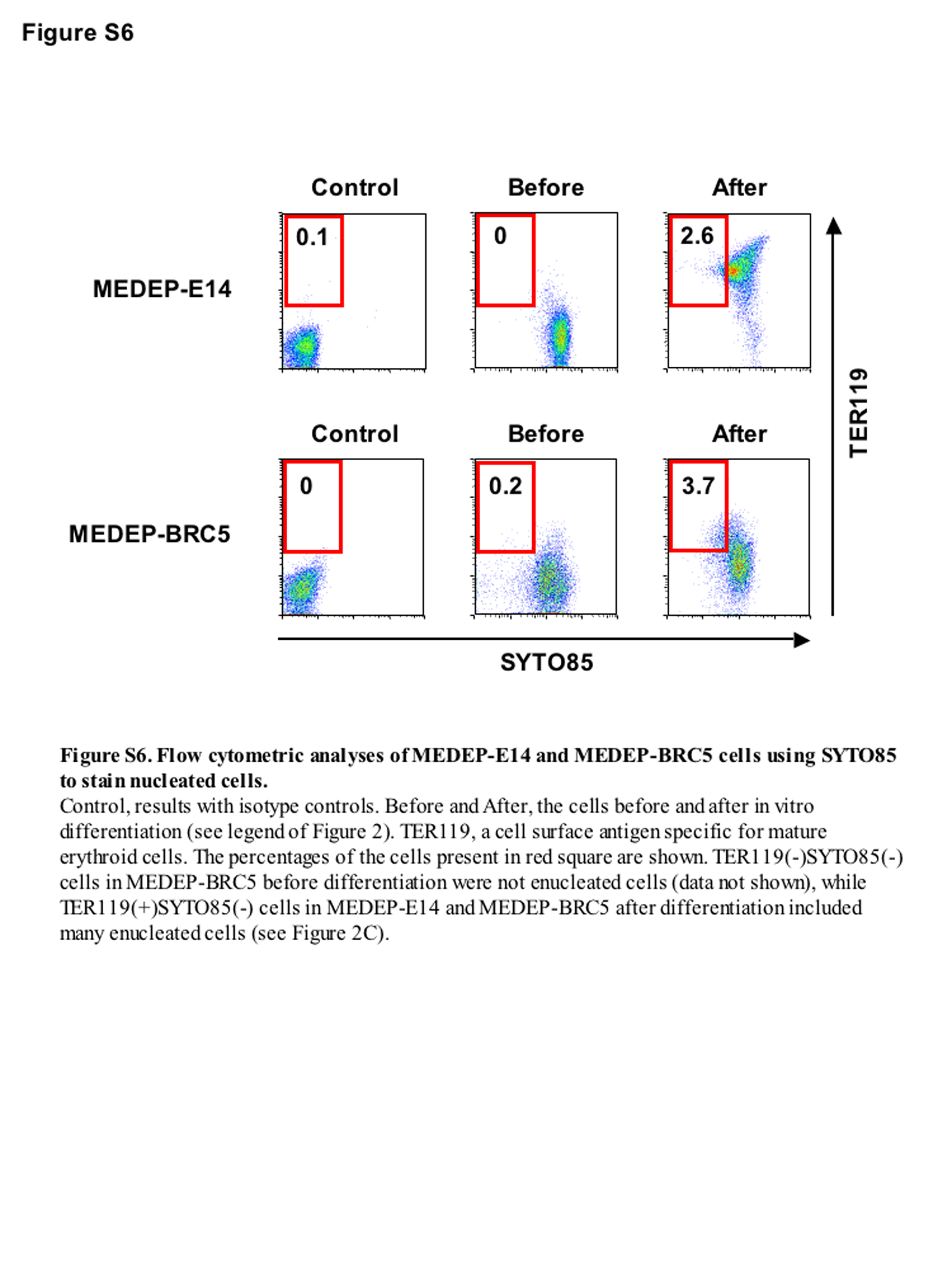

Supplement: Figure S6 — (10.19 MB TIF) [file pone.0001544.s006.tif]

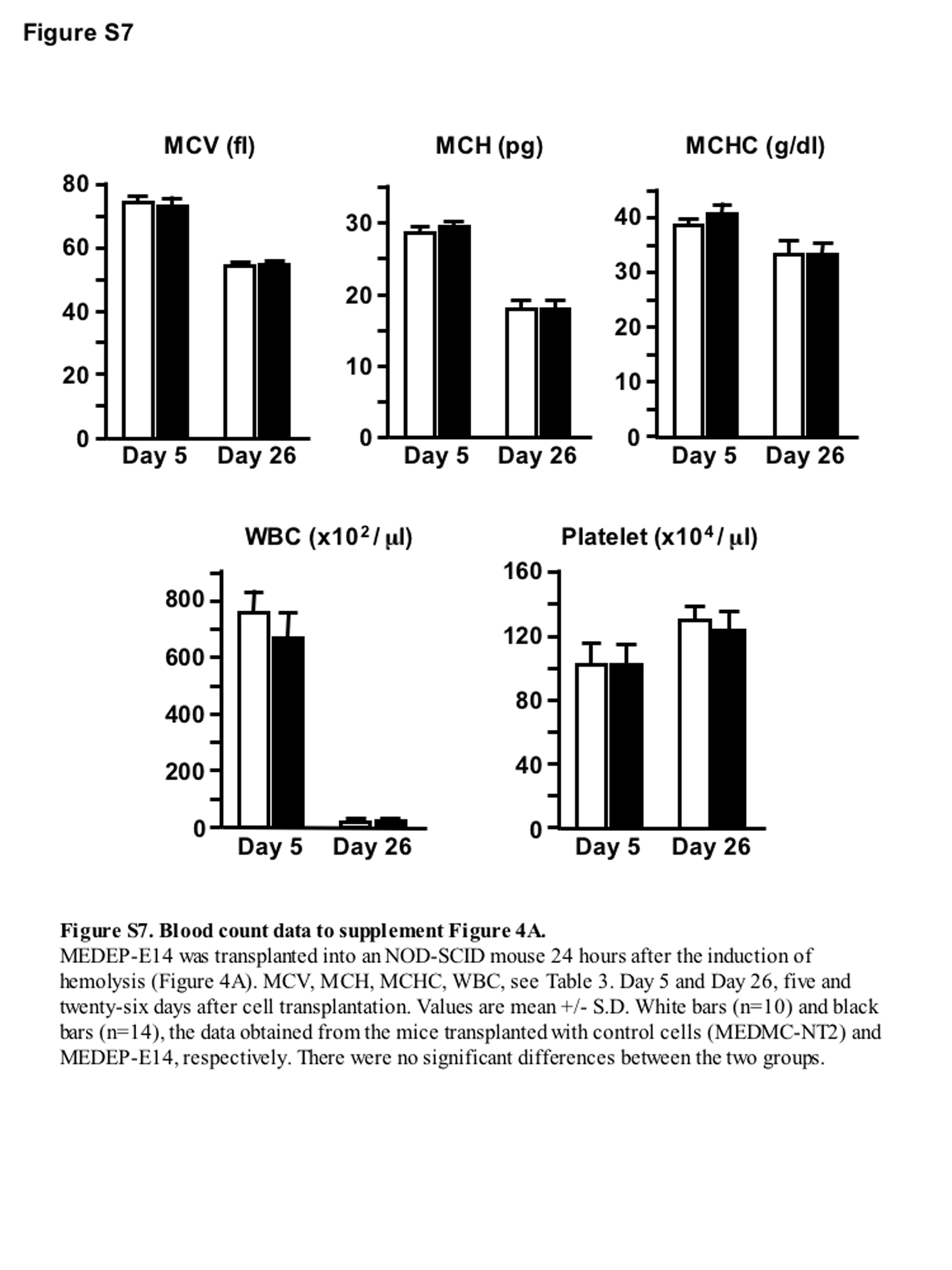

Supplement: Figure S7 — (10.19 MB TIF) [file pone.0001544.s007.tif]

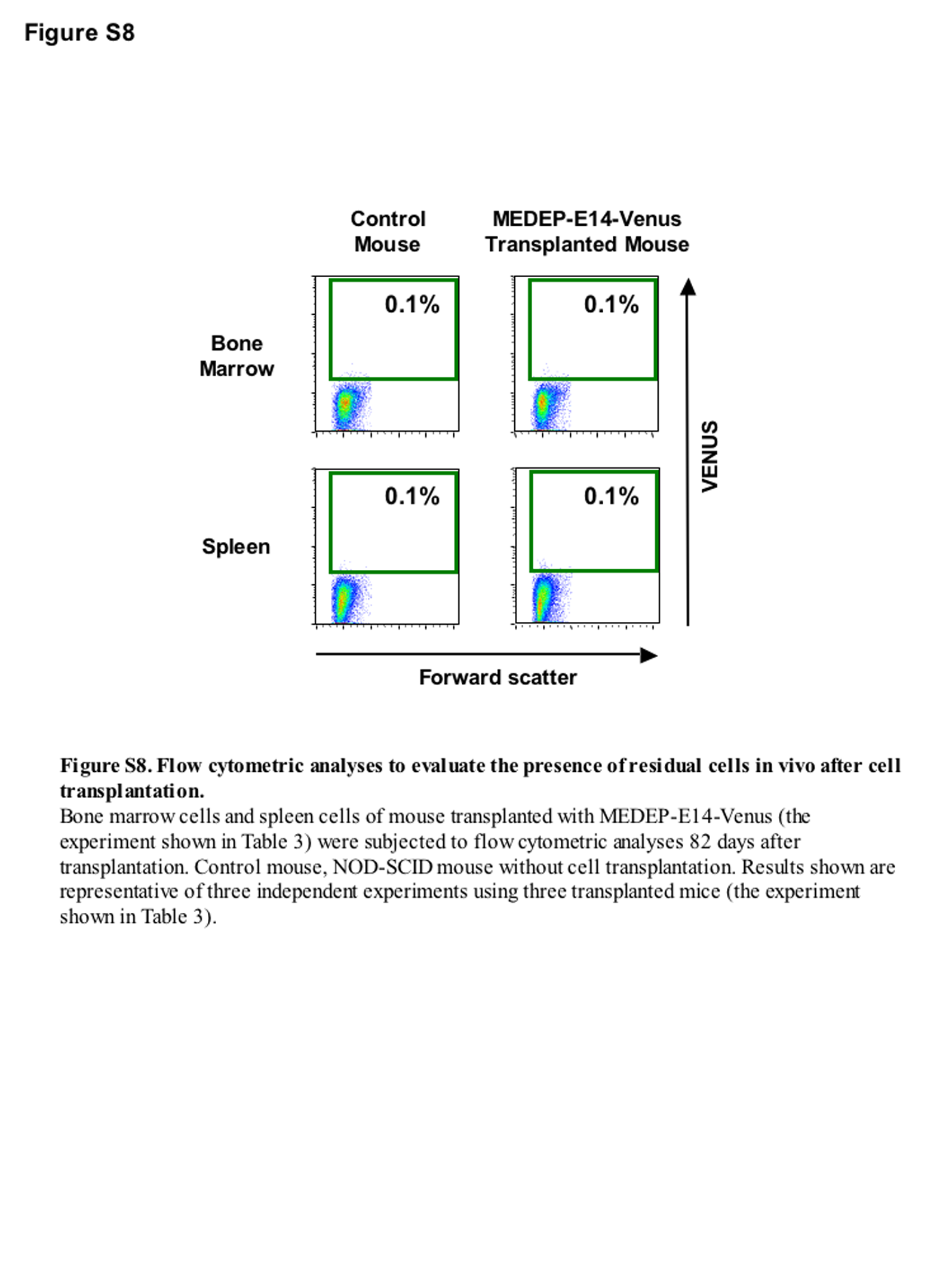

Supplement: Figure S8 — (10.19 MB TIF) [file pone.0001544.s008.tif]
